# The Role of SMAD2/3 in Human Embryonic Stem Cells

**DOI:** 10.3389/fcell.2020.00653

**Published:** 2020-07-21

**Authors:** Jie Yang, Wei Jiang

**Affiliations:** ^1^Department of Biological Repositories, Frontier Science Center for Immunology and Metabolism, Medical Research Institute, Zhongnan Hospital of Wuhan University, Wuhan University, Wuhan, China; ^2^Hubei Provincial Key Laboratory of Developmentally Originated Disease, Wuhan University, Wuhan, China; ^3^Human Genetics Resource Preservation Center of Wuhan University, Wuhan, China

**Keywords:** SMAD2/3, human embryonic stem cell, pluripotency, cell cycle, differentiation, lncRNAs, epigenetic modification

## Abstract

Human embryonic stem cells (hESCs) possess the potential of long-term self-renewal and three primary germ layers differentiation, and thus hESCs are expected to have broad applications in cell therapy, drug screening and basic research on human early embryonic development. Many efforts have been put to dissect the regulation of pluripotency and direct differentiation of hESCs. TGFβ/Activin/Nodal signal pathway critically regulates pluripotency maintenance and cell differentiation through the main signal transducer SMAD2/3 in hESCs, but the action manners of SMAD2/3 in hESCs are sophisticated and not documented yet. Here we review and discuss the roles of SMAD2/3 in hESC pluripotency maintenance and differentiation initiation separately. We summarize that SMAD2/3 regulates pluripotency and differentiation mainly through four aspects, (1) controlling divergent transcriptional networks of pluripotency and differentiation; (2) interacting with chromatin modifiers to make the chromatin accessible or recruiting METTL3-METTL14-WTAP complex and depositing m6A to the mRNA of pluripotency genes; (3) acting as a transcription factor to activate endoderm-specific genes to thus initiate definitive endoderm differentiation, which happens as cyclin D/CDK4/6 downstream target in later G1 phase as well; (4) interacting with endoderm specific lncRNAs to promote differentiation.

## Introduction

The TGF-β superfamily comprises TGF-βs, activins, nodal, growth and differentiation factors (GDFs) and bone morphogenetic proteins (BMPs). More than 60 TGF-β family members have been identified in multicellular organisms, with at least a half of proteins are encoded in human genome (Feng and Derynck, 2005). TGF-β signal originates from the binding of ligand dimers and heteromeric complex of type I (ALK1-7) and type II transmembrane serine/threonine kinase receptors. Activated type II receptors phosphorylate the type I receptors kinase domain, and then phosphorylate the intracellular SMAD proteins (Wu and Hill, 2009). There are three functional classes containing eight SMAD proteins: receptor-regulated SMADs (R-SMAD) including SMAD1/2/3/5/8, Co-mediator SMAD4 (Co-SMAD), and the inhibitory SMAD6 and 7 (I-SMAD). All SMAD proteins share Mad Homology domains MH1 and MH2: the MH1 domain contains a β-hairpin structure that mediates DNA binding, and the MH2 domain mediates SMAD oligomerization and establishes signal specificity by mediating proper type I receptor interaction with specific DNA binding partners (Weiss and Attisano, 2013). TGF-β/Activin/Nodal signaling occurs through ALKs 4, 5, and 7 and SMAD2/3 (Massague et al., 2005). It is reported that inhibition of Activin/Nodal signaling causes hESCs differentiation (James et al., 2005; Vallier et al., 2005; Xiao et al., 2006). On the other hand, Activin/Nodal signaling, through the TGF-β receptors and its effector SMAD2/3, initiates definitive endoderm differentiation in human and mouse ESC (Tremblay et al., 2000; Kubo et al., 2004; D’Amour et al., 2005). Accumulated reports show that TGF-β/Activin/Nodal signaling and its effectors SMAD2/3 are not only necessary for hESC self-renewal but also required for germ layer differentiation. However, the mechanism that SMAD2/3 coordinates different partners to determine different cell fate decisions is not systematically elaborated. Herein, this review is focusing on the roles and mechanisms of SMAD2/3 in regulating hESC pluripotency maintenance and germ layer differentiation.

## SMAD2/3 Controls Divergent Transcriptional Networks of Pluripotency and Endoderm Differentiation

**FIGURE 1:**
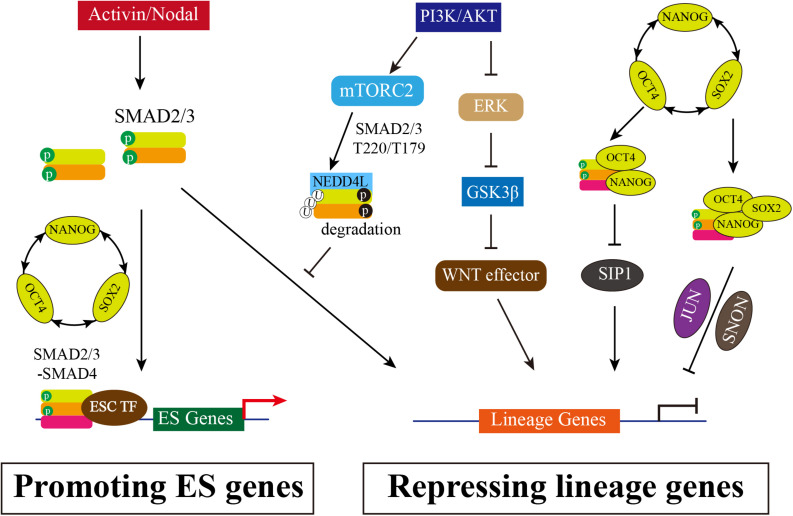
The Multiple Roles of SMAD2/3 in Maintenance of hESCs. Activated TGF-β/Activin/Nodal signaling causes SMAD2/3 phosphorylation in distal C-terminal SXS motif (in green), which thus interacts with SMAD4 to form heterooligomeric complex and enters nucleus. Accumulated SMAD2/3/4 complex in nucleus can directly bind to the promotor regions of pluripotency marker genes, such as *NANOG* and *OCT4*, promoting the positive feedback loop of OCT4-SOX2-NANOG. In addition, SMAD2/3 can inhibit SIP1 expression to block neuroectodermal differentiation. SMAD2/3 also interacts with NANOG to repress differential genes expression, or recruits SNON activated by OCT4/SOX2/NANOG, or interacts with JUN, and then together with OCT4/SOX2/NANOG complex to suppress lineage genes expression. Meanwhile, PI3K/AKT is highly activated in hESCs. PI3K/AKT on one hand inhibits ERK and WNT signaling, allowing SMAD2/3 to activate pluripotency associated target genes rather than lineage genes; on the other hand, the downstream of PI3K/AKT mTORC2 can phosphorylate SMAD2/3 resulting the degradation mediated by NEDD4Land thus blocking the direct activation of lineage genes.

Human ESCs are derived from the blastocyst with the potential of long-term self-renewal and three primary germ layers differentiation (Thomson et al., 1998) and cultured in medium with basic fibroblast growth factor (bFGF) and Activin or TGF-β. TGF-β/Activin/Nodal signaling is essential for the maintenance of hESC pluripotency and self-renewal, while FGF2 serving as a competence factor (James et al., 2005; Xiao et al., 2006). Inhibition of Activin/Nodal signaling by Follistatin or by Activin receptor inhibitor SB431542 causes hESC differentiation (James et al., 2005; Vallier et al., 2005; Xiao et al., 2006). Pluripotency is maintained by the OCT4-SOX2-NANOG transcriptional network, which forms a positive feedback loop that activates pluripotency genes and inhibits the expression of differentiation associated genes (Boyer et al., 2005; He et al., 2009). Activin A is sufficient for the maintenance of self-renewal and pluripotency and induces the expression of OCT4, NANOG and SOX2 (Xiao et al., 2006; Xu et al., 2008), suggesting that pluripotent transcription factors might be the targets of TGF-β/Activin/Nodal signaling in hESCs. Inhibition of Activin/Nodal results in a more decrease of NANOG expression than OCT4 and SOX2 in hESCs, and thus induces differentiation toward neuroectoderm. Constitutive expression of NANOG is sufficient to maintain the pluripotency of hESC in the absence of Activin/Nodal signaling (Vallier et al., 2009a, b). In addition, transfection of small interfering RNAs targeting *NANOG* in hESCs causes differentiation to extraembryonic endoderm and trophectoderm lineages (Hyslop et al., 2005). Therefore, NANOG is considered as a direct target of TGF-β/Activin/Nodal signaling, which is further demonstrated by the study that SMAD2/3 can directly bind with the NANOG proximal promoter to activate NANOG expression and repress autocrine BMP signaling (Xu et al., 2008; Vallier et al., 2009a; Brown et al., 2011; Sakaki-Yumoto et al., 2013). Meanwhile, NANOG and SMAD2/3 can bind to regulatory elements of endoderm genes with other transcription repressors to inhibit differentiation (Xu et al., 2008; Brown et al., 2011). For instance, NANOG and SMAD2/3 inhibits Smad-interacting protein 1 (SIP1) expression to block neuroectoderm differentiation (Chng et al., 2010). Like SIP1, SNON (also named as SKIL), a potent SMAD2/3 corepressor, is expressed in hESCs but rapidly down-regulated upon differentiation. In pluripotent state, SNON is transcriptionally activated by OCT4/SOX2/NANOG complex and is selectively recruited by SMAD2, and then SMAD2/SNON together binds to mesendodermal genes to suppress the expression (Tsuneyoshi et al., 2012). A recent report shows that Jun N-terminal kinase (JNK)-JUN family genes JUN co-occupies ESC-specific enhancers with OCT4, NANOG, SMAD2/3, and specifically inhibits the exit from the pluripotent state by impeding the decommissioning of ESC enhancers and inhibiting the reconfiguration of SMAD2/3 chromatin binding from ESC-specific to endoderm-specific enhancers (Li et al., 2019). Overall, SMAD2/3 is necessary for self-renewal and pluripotency maintenance: SMAD2/3 not only targets NANOG to positively regulate OCT4-SOX2-NANOG transcriptional network, but also interacts with repressors such as SIP1, SNON and JUN to inhibit lineage genes associated with primary germ layer differentiation (Figure 1).

However, highly activated Activin/Nodal signal results in definitive endoderm differentiation. High concentrations of Activin A induces efficient differentiation of hESCs towards definitive endoderm: 50–100 ng/ml Activin A drives endoderm differentiation (D’Amour et al., 2005), whereas 5 ng/ml Activin A is supportive to maintain pluripotency of hESCs (Xiao et al., 2006; Tsai et al., 2010; Tomizawa et al., 2013). SMAD2/3 directly binds to endodermal lineage specifiers such as *SOX17*, *FOXA2*, *GATA6*, and *GSC* to induce endoderm differentiation (Brown et al., 2011; Kim et al., 2011). In addition, the pluripotent factors OCT4, SOX2, and NANOG control EOMES expression at onset of endoderm specification, and SMAD2/3 further interacts with EOMES to initiate endodermal transcription network expression (Teo et al., 2011). Activin/Nodal signaling also interacts with other signal pathways, mainly including BMP and WNT to synergistically promote endoderm differentiation (D’Amour et al., 2005; Loh et al., 2014). Higher activity of WNT/β-catenin stimulated by high dosage of WNT3A or glycogen synthase kinase-3 (GSK-3) inhibitors is required for maximally inducing definitive endoderm differentiation together with Activin A (Gadue et al., 2006; Teo et al., 2014). PI3K/AKT signaling, stimulated by fibroblast growth factor (FGF), directly modulates the downstream of WNT pathway to maintain undifferentiated hESCs (Ding et al., 2010). PI3K/AKT can also inhibit SMAD3 phosphorylation causing blocking of nuclear translocation (Remy et al., 2004). In addition, the inhibition of PI3K activity causes decrease of the mRNA and protein levels of Nanog (Storm et al., 2007), and activation of PI3K/AKT signaling is sufficient to maintain pluripotency of primate ESCs (Watanabe et al., 2006). Furthermore, Activin/Nodal specifies definitive endoderm from hESCs only when PI3K signaling is low (McLean et al., 2007). It seems that PI3K/AKT signaling is the important switcher for Activin/Nodal signaling in pluripotency or differentiation. Supporting with this notion, a later study suggests that PI3K governs Activin A/SMAD2/3 to promote pluripotency or differentiation cell fate in hESCs when PI3K activity is in high or low level, respectively (Singh et al., 2012). Singh and colleagues found that PI3K/AKT inhibits RAF/MEK/ERK and canonical WNT signaling, allowing SMAD2/3 to activate pluripotency associated target genes, such as NANOG, at high level of PI3K in undifferentiated state. When PI3K/AKT signaling is absent, the ERK activates canonical WNT signaling pathways and then the WNT effectors such as β-catenin and SNAIL can permit SMAD2/3 to activate differentiation associated genes (Singh et al., 2012). However, there is a controversial: Na and colleagues observed the inhibition of MEK/ERK prevents differentiation and promotes hESCs self-renewal (Na et al., 2010) while others showed that MEK/ERK signaling positively contributes to maintain self-renewal of hESCs (Armstrong et al., 2006; Li et al., 2007). Yu and colleagues provide more details in molecular mechanism. PI3K antagonizes the Activin/Nodal signaling in definitive endoderm differentiation by activated rapamycin complex 2 (mTORC2). Activation mTORC2 induces SMAD2/3 phosphorylation at T220/T179 residues which is not dependent on AKT, CDK, or ERK activity. The phosphorylated SMAD2/3 recruits E3 ubiquitin ligase NEDD4L, which causes SMAD2/3 degradation and blocks endoderm differentiation (Yu et al., 2015).

Taken together, Activin/Nodal signaling via SMAD2/3 on one hand is involved in controlling divergent transcriptional networks by interacting with different partners to regulating pluripotency or differentiation in hESCs. On the other hand, Activin/Nodal signaling antagonizes with PI3K/AKT pathway in pluripotency and differentiation. High PI3K and low Activin activity in hESCs promotes SMAD2/3 binding and activating gene expression of pluripotent transcription factors (Figure 1).

## SMAD2/3 Interacts With Epigenetic Modifiers

**FIGURE 2:**
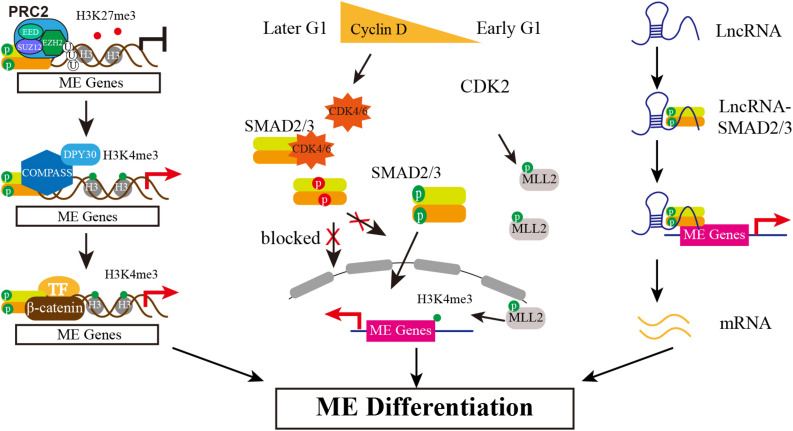
The Multiple Mechanisms of SMAD2/3 Regulating Mesendoderm Differentiation of hESCs. Epigenetically, SMAD2/3 recruits JMJD3 to its target genes, erasing H3K27me3 marker. Then, SMAD2/3 interacts with COMPASS and its cofactor DPY30 to maintain H3K4me3 marks on these genes. In addition, differentially expressed Cyclin D in early and later G1 phase determines the biased lineage differentiation propensity. Low expression of Cyclin D in early G1 causes low level of CDK4/6, blocking the phosphorylation of SMAD2/3 in the linker region (in red) and thus allowing SMAD2/3 enter nucleus to activate mesendodermal genes expression. Meanwhile, CDK2 may phosphorylate MLL2 during G1 and causes H3K4me3 transiently increased at developmental genes, thereby opening a “window of opportunity” for lineage specification. The third action model is mediated by lncRNAs. Certain highly expressed lncRNAs interact with SMAD2/3 and recruit SMAD2/3 to and activate the expression of endodermal transcription factors such as *FOXA2*/*GSC*.

The pluripotency and differentiation of ESC is regulated not only by the core transcriptional network but also epigenetic modifiers, as epigenetic modification supports a permissive or repressive chromatin environment for transcription. In mouse ESCs, SMAD2/3 recruits the histone demethylase JMJD3 to target genes *Nodal* and *Brachyury*, thereby counteracting repression of Polycomb (Dahle et al., 2010). A follow-up study reveals that SMAD2/3 can also counteract Polycomb repression to regulate *Oct4* expression during initiation of ESC differentiation (Dahle and Kuehn, 2013). Similarly, helix-loop-helix (HLH) proteins HEB also interacts with SMAD2/3 at distal enhancer elements and associates with PRC2 at promotors of mesendodermal genes (Yoon et al., 2015). In addition, it is reported that TGF-β/Nodal signals trigger differentiation in mouse ESC by influencing H3K9me3 modification, the hallmark of heterochromatin. The activation of Nodal signal induces the formation of TRIM33-SMAD2/3 complex. The PHD and Bromo cassette of TRIM33, respectively, recognizes K9me3 and binds an adjacent K18ac, making the chromatin accessible, which in turns allows SMAD2/3-SMAD4 to bind to the promotor of *Gsc* and *Mixl1* to finally promote stem cell differentiation (Xi et al., 2011). Whether there is similar mechanism that SMAD2/3 interacts with Polycomb complex to respond to pluripotency maintenance or differentiation initiation in hESCs is rarely reported until recent years. Wang and colleagues show that in hESCs Activin signal impairs PRC2 activity by SMAD2-mediate reduction of EZH2 protein level, which is the catalytic subunit of PRC2 (Wang et al., 2017). Further study documents that the global reduction of H3K27me3 by Activin signal causes the forkhead protein FOXH1 to recruit into open chromatin regions, which together with SMAD2 and β-catenin activates mesendodermal genes expression, such as HAS2 and ALDH3A2 (Xu et al., 2018). Except for inhibitory epigenetic modification, it is also reported that Activin/Nodal signaling is relevant with the H3K4me3 maintenance on the master regulators of both pluripotency and germ layer specification in hESCs (Bertero et al., 2015). SMAD2/3 interacts with H3K4 methyltransferases complex COMPASS and its cofactor DPY30 to maintain H3K4me3 marks on pluripotency and mesendoderm genes. Inhibiting Activin/Nodal signaling causes specifically impaired H3K4me3 level on promotor and enhancer regions. Knockdown DPY30 in hESCs reduces expression of pluripotency and endoderm markers but increases neuroectoderm genes. Further mechanistic studies show that SMAD2/3 cooperates with NANOG and DPY30 to regulate H3K4me3 deposition on pluripotency and cell fate decision associated genes (Bertero et al., 2015). Recently the same group shows SMAD2/3 interacts with the METTL3-METTL14-WTAP complex identified by genome-wide analysis of SMAD2/3 interactome, which deposits N6-methyladenosine (m6A) on RNA. The interaction between SMAD2/3 and METTL3-METTL14-WTAP complex promotes m6A deposition on specific regulators of pluripotency such as NANOG, which resulting the degradation of NANOG mRNA and thus facilitating pluripotency exit and differentiation initiation (Bertero et al., 2018). Taken together, SMAD2/3 can interact with multiple epigenetic modifiers to function in different levels, together controlling pluripotency and mesendoderm differentiation initiation (Figure 2).

## SMAD2/3 Mediates hESC Differentiation Propensity in G1 Phase

Human ESCs in different cell cycle phases exhibit biased differentiation propensity, which involves in CDK4/6-mediated SMAD2/3 phosphorylation. Primate ESCs exhibit unusual cell cycle features which are different from mouse ESCs, including dramatically shortened G1 phase (Becker et al., 2006; Fluckiger et al., 2006). Subsequent studies show that shortened G1 phase of cell cycle in hESCs is a cause, rather than a consequence of pluripotency. A short G1 limits the “window of opportunity” for which a cell can be responsive to differentiation cues (Neganova et al., 2009; Lange and Calegari, 2010), and lengthening G1 phase of pluripotent cell promotes differentiation in mouse and human (Filipczyk et al., 2007; Koledova et al., 2010; Lange and Calegari, 2010; Sela et al., 2012). Human ESCs express all G1-specific Cyclins (D1, D2, D3, and E) and cyclin-dependent kinases (CDK) (CDK2, CDK4, and CDK6) at variable levels (Neganova et al., 2009; Lange and Calegari, 2010). Knockdown of CDK2 in hESCs results in arrest at G1 phase and differentiation to extraembryonic (Neganova et al., 2009), suggesting that CDK2 is very important for cell cycle regulation and pluripotency maintenance in hESCs. Recent reports have provided some explanations for how to determine cell fate propensity in G1 phase. Using FUCCI reporter system, which is a sensor of cell cycle, Pauklin and Vallier observed hESCs in early G1 phase can only initiate endoderm differentiation but neuroectoderm differentiation is limited in later G1 phase. Further mechanistic analysis shows that the activity of Activin/Nodal signaling is controlled by Cyclin D, which activates CDK4/6 to phosphorylate SMAD2/3 in the linker region and thus makes the phosphorylated fail to enter nucleus. Since the Cyclin D expression is low in early G1 and high in later G1, thus Cyclin D/CDK4/6 inhibits the transcriptional activity of Activin/Nodal signaling to determine cell fate propensity by controlling the cellular localization of SMAD2/3 (Pauklin and Vallier, 2013). Consistently, SMAD3 is proved to be the substrate of CDK4 in MEF, and the phosphorylated SMAD3 by CDK4 inhibits its transcriptional activity (Matsuura et al., 2004).

Singh and colleagues also reported the heterogeneity correlated with cell cycle, but their results indicate that WNT/ERK signal promotes heterogeneity in late G1 cells, including developmental regulators expression such as *GATA6*, *SOX17*, *FOXA2* (Singh et al., 2013). A later study by the same group further proved that the bivalent state is not stable in hESCs either, especially in G1 phase. H3K4me3 is transient increased at developmental genes by CDK2-dependent phosphorylation of the MLL2 histone methyl-transferase during G1, thereby opening a “window of opportunity” for lineage specification (Singh et al., 2015). In addition, a recent single-cell level study shows that hESCs exhibit high single-cell variation in absolute G1 length which is controlled by WNT/β-catenin pathway. A longer and wider distribution of G1 phase is regulated by WNT inhibition, allowing global reduction of 5-hydroxymethylcytosine (5hmC) on lineage-specific genes and thus causing biased differentiation toward neuroectoderm lineages but not affecting pluripotent genes expression. Transient decrease of G1 length by transgenic manipulation shows predominantly contributing to mesendoderm (Jang et al., 2019). Different with the previous report (Pauklin and Vallier, 2013), the single-cell analysis results show that the length of G1 has no effect on SMAD2/3 activity (Jang et al., 2019). Taken together, it is consistent that G1 phase creating a “window of opportunity” for mesendodermal genes expression, which endows hESC differentiation propensity to mesendoderm in early G1 phase (Figure 2). However, how to initiate lineage specification and whether SMAD2/3 is controlled in G1 phase are still controversial and need to be further clarified.

## SMAD2/3 Interacts With lncRNA Promoting Differentiation

Long non-coding RNAs (lncRNAs) are transcribed with longer than 200 nucleotides and have been found wildly expressed from mammal genome, and play an important role in diverse biological processes, including regulation of stem cell (Fatica and Bozzoni, 2014). During hESCs differentiates into endoderm, there are reported that SMAD2/3 serve as RNA-interacting protein to actively participate in endoderm differentiation by different action mechanism (Jiang et al., 2015; Daneshvar et al., 2016; Chen et al., 2020). *DEANR1* is the first reported lncRNA that regulates human definitive endoderm differentiation. *DEANR1* is highly expressed in endoderm and contributes to endoderm differentiation by positively regulating endoderm factor FOXA2. Mechanistically, *DEANR1* facilitates FOXA2 activation by interacting with SMAD2/3 and recruiting to the FOXA2 promotor (Jiang et al., 2015). *DIGIT*, an endoderm-expressing lncRNA, is regulated by a SMAD3-occupied enhancer proximal to *DIGIT*. *DIGIT* regulates the transcription of *GSC in trans*, and deletion of the SMAD3-occupied enhancer inhibits *DIGIT* and *GSC* expression and definitive endoderm differentiation (Daneshvar et al., 2016). Very recently, lncRNA *LINC00458* is reported up-regulated in hESCs upon cultured with soft substrate. Gain- and loss-of-function experiments confirm that *LINC00458* is functionally required for endodermal lineage specification from hESCs induced by soft substrates. Importantly, the endoderm-promoting function of *LINC00458* depends on the interaction with SMAD2/3 (Chen et al., 2020). These reports together suggest a new model that lncRNA may function as a new co-factor of SMAD2/3 to mediate lineage differentiation (Figure 2).

## Summary and Prospect

SMAD2/3 is the major effector of TGFβ/Activin/Nodal signal, which plays different roles in undifferentiated hESCs and committed cells. Herein, we summarize the multiple action manners of SMAD2/3 in pluripotency maintenance and differentiation initiation in terms of transcription regulation, epigenetic modification, cell cycle related differentiation bias and interaction with lncRNAs. SMAD2/3 protects pluripotent state by directly targeting OCT4-SOX2-NANOG network to maintain pluripotency and interacts with other factors such as SIP1, SNON, and JUN to inhibit differentiation at high PI3K/AKT activity. When in differentiation context, SMAD2/3 is highly activated by higher Activin/Nodal signal while low PI3K/AKT activity. Accessible chromatin due to the increase of 5hmC and H3K4me3 allows SMAD2/3 binding to developmental regulators to perform cell-fate decision function. It is very likely that Activin/Nodal and its antagonistic PI3K/AKT signal together determine SMAD2/3 functional switch in pluripotency and differentiation. In addition, WNT pathway synergies with Activin/Nodal to permit SMAD2/3 to activate differentiation associated genes. Meanwhile, the co-regulators of SMAD2/3 also are decisive for SMAD2/3 function in different contexts. However, there are controversies about the role of SMAD2/3 in cell cycle dependent differentiation initiation. In addition, how SMAD2/3 chooses different co-regulators during the pluripotency maintenance and differentiation transition is still unclear but very important to understand the underlying mechanism of cell fate determination.

Furthermore, the SMAD2/3 interactome analysis suggests SMAD2/3 interacts with many other functional complexes except for classic TGF-β signaling associated pathways, including E3 ubiquitin ligase, mRNA processing and degradation, DNA repair and apoptosis (Bertero et al., 2018). Whether or which of these interactions with SMAD2/3 are important for ESCs pluripotency maintenance or differentiation is not uncovered yet. In addition, SMAD2/3 also participates in lipid metabolism in mouse hepatocytes (Yang et al., 2013) and epithelial-mesenchymal transition and metastasis induction in breast cancer (Rios Garcia et al., 2017). Of note, there is significant metabolic difference between pluripotent state and differentiation in stem cells (Mathieu and Ruohola-Baker, 2017). Does SMAD2/3 also directly regulate metabolism related protein or signal pathways to influence pluripotency or differentiation in hESCs? In addition to the roles of SMAD2/3 reviewed in this article, there are still many unknown roles of SMAD2/3 in hESCs that need to be clarified.

## Author Contributions

JY conceived the project and drafted the manuscript. WJ and JY prepared the figures and finalized the manuscript. Both authors contributed to and approved the final manuscript.

## Conflict of Interest

The authors declare that the research was conducted in the absence of any commercial or financial relationships that could be construed as a potential conflict of interest.
